# Clinical and molecular characteristics of East Asian patients with von Hippel–Lindau syndrome

**DOI:** 10.1186/s40880-016-0141-z

**Published:** 2016-08-15

**Authors:** Meihua Wong, Ying-Hsia Chu, Hwei Ling Tan, Hideharu Bessho, Joanne Ngeow, Tiffany Tang, Min-Han Tan

**Affiliations:** 1 Division of Medical Oncology, National Cancer Centre Singapore, Singapore, 169610 Singapore; 2 Institute of Bioengineering and Nanotechnology, Singapore, 138669 Singapore; 3 Department of Urology, Kitasato University School of Medicine, Sagamihara, Kanagawa 252-0374 Japan

**Keywords:** Von Hippel–Lindau syndrome, Renal cell carcinoma, Retinal capillary hemangioblastoma

## Abstract

**Background:**

Von Hippel–Lindau (VHL) syndrome is a dominantly inherited multisystem cancer syndrome caused by a heterozygous mutation in the *VHL* tumor suppressor gene. Previous studies suggested that similar populations of Caucasian and Japanese patients have similar genotype or phenotype characteristics. In this comprehensive study of East Asian patients, we investigated the genetic and clinical characteristics of patients with VHL syndrome.

**Methods:**

To create a registry of clinical characteristics and mutations reported in East Asian patients with VHL syndrome, we conducted a comprehensive review of English language and non-English language articles identified through a literature search. Publications in Japanese or Chinese language were read by native speakers of the language, who then performed the data extraction.

**Results:**

Of 237 East Asian patients with VHL syndrome, 154 unique kindreds were identified for analysis. Analyzed by kindred, missense mutations were the most common (40.9%, 63/154), followed by large/complete deletions (32.5%, 50/154) and nonsense mutations (11.7%, 18/154). Compared with a previously reported study of both East Asian and non-East Asian patients, we found several key differences. First, missense and frameshift mutations in the *VHL* gene occurred less commonly in our population of East Asian patients (40.9% vs. 52.0%; *P* = 0.012 and 8.4% vs. 13.0%; *P* < 0.001, respectively). Second, large/complete deletions were more common in our population of East Asian patients (32.5% vs. 10.5%; *P* < 0.001). Third, phenotypically, we observed that, in our population of East Asian patients with VHL syndrome, the incidence of retinal capillary hemangioblastoma was lower, whereas the incidence of renal cell carcinoma was higher.

**Conclusions:**

Evidence suggests that the genotypic and phenotypic characteristics of East Asian patients with VHL syndrome differ from other populations. This should be considered when making screening recommendations for VHL syndrome in Asia.

**Electronic supplementary material:**

The online version of this article (doi:10.1186/s40880-016-0141-z) contains supplementary material, which is available to authorized users.

## Background

Von Hippel–Lindau (VHL) syndrome is an inherited multisystem cancer syndrome with an estimated incidence of 1 in 36,000 live births [1]. Those affected are predisposed to a spectrum of benign and malignant tumors of the central nervous system, retina, kidney, adrenal gland, pancreas, reproductive adnexal organ, and inner ear [2, 3].

Von Hippel–Lindau syndrome is transmitted in an autosomal dominant fashion and has a penetrance reported to exceed 90% by 60 years of age [4]. It is caused by germline mutations in the *VHL* tumor suppressor gene, located on the short arm of chromosome 3. The gene consists of 3 exons and encodes 2 protein products: a full-length 30-kDa form (p30) composed of 213 amino acids and a 19-kDa form (p19). The VHL protein (pVHL) is the substrate-binding subunit of an E3 ubiquitin ligase complex and has an alpha domain and a beta domain [5]. Through its beta domain, pVHL binds directly to hypoxia-inducible factor (HIF) [6] and targets the HIF alpha subunit for oxygen-dependent proteolysis [7].

Interfamilial differences in pheochromocytoma (PCC) susceptibility have been recognized, and a clinical classification of VHL syndrome was created based on the risk of developing PCC: VHL patients with type 1 disease are at low risk, whereas those with type 2 disease are at high risk [8, 9]. The cancer phenotypes of type 2 disease have been further subdivided based on the likelihood of developing renal cell carcinoma (RCC): type 2A and type 2B have a low and high likelihood, respectively; patients with type 2C disease develop PCCs only, without developing RCCs or hemangioblastomas [2]. Over 900 mutations of the *VHL* gene have been described [10]. The mutations accounting for type 1 disease include missense mutations (43%), frameshift mutations (17%), nonsense mutations (13%), partial/complete deletions (10%), splice (9%), and in-frame deletion/insertions (8%) [11]. Most mutations associated with type 2 disease are missense mutations [9, 11].

In the present study, we consolidated published data on genetic and clinical information about VHL syndrome in Asia to further understand the spectrum of VHL syndrome, focusing on differences between East Asian and Western patient populations.

## Patients and methods

### Local patient selection

Patients were identified through the clinical cancer genetics service in the National Cancer Centre Singapore. Before informed consent was provided to obtain blood samples for genetic testing, patients were provided genetic counseling by specialized clinical cancer geneticists. Patients and their families with confirmed *VHL* mutations were selected for the study and registered in a database. Institutional review board approval was obtained for this study.

### Literature search strategy

On June 15, 2012, we performed a systematic search on PubMed using the following search strategy: “(von Hippel Lindau Disease OR Familial Cerebello-Retinal Angiomatosis OR Angiomatoses, Familial Cerebello-Retinal OR Angiomatosis, Familial Cerebello-Retinal OR Cerebello-Retinal Angiomatoses, Familial OR Cerebello-Retinal Angiomatosis, Familial OR Familial Cerebello Retinal Angiomatosis OR Familial Cerebello-Retinal Angiomatoses OR von Hippel–Lindau Syndrome OR Syndrome, von Hippel–Lindau OR von Hippel Lindau Syndrome OR Lindau Disease OR Lindau’s Disease OR Lindau’s Diseases OR Lindaus Disease OR Cerebelloretinal Angiomatosis, Familial OR Angiomatoses, Familial Cerebelloretinal OR Angiomatosis, Familial Cerebelloretinal OR Cerebelloretinal Angiomatoses, Familial OR Familial Cerebelloretinal Angiomatoses OR Familial Cerebelloretinal Angiomatosis OR Hippel–Lindau Disease OR Hippel Lindau Disease) AND (China OR Hong Kong OR Chinese OR Japan OR Japanese OR Korea OR Korean OR Taiwan OR Republic of China OR Taiwanese).”

All available articles were reviewed and included for analysis if both genotype and phenotype information were reported. Articles in the Chinese and Japanese languages were reviewed by native speakers. All data were input into a standardized template. We extracted the following data: (i) genetic information—sequence variation at the DNA and protein levels [12], and type of variation (e.g., missense); (ii) clinical information—diagnosis of central nervous system hemangioblastoma, retinal capillary hemangioblastoma (RCH), PCC, pancreatic cyst or tumor, renal cyst or tumor, endolymphatic sac tumor, or papillary cystadenoma of the epididymis or broad ligament; (iii) patient demographics—age at first diagnosis, sex, nationality, race, and familial or sporadic inheritance; and (iv) article publication data—journal, year of publication, and authors.

The open source program Mutalyzer was used to check the protein consequences of mutations [13]. To avoid duplication of data, if the same sequence variation was reported at the DNA level, we compared patient nationality, sex, age of first diagnosis, and disease manifestations to determine the likelihood of duplicate data. The entire dataset is included in the Additional file 1: Table S1.

### Statistical analysis

The data from all VHL families were analyzed (Additional file 1: Table S1). SPSS 16.0 software (SPSS Inc., Chicago, IL, USA) was used for statistical analysis. Clinical characteristics were compared with the Chi square test, and *P* values less than 0.05 were considered statistically significant.

## Results

### Literature review

The described search strategy of VHL syndrome in an East Asian population identified 347 articles. Of the total of 237 East Asian VHL patients, 154 kindreds were identified for analysis based on available genotype and phenotype information.

### Local patient selection

Ten affected individuals from 7 families in Singapore were identified.

### Analysis of mutations

In our study of East Asian patients, we calculated the frequency of each mutation and then compared this with the dataset reported by Nordstrom-O’Brien et al. [11], which included both Asian and non-Asian patients. Analyzed by similarity, the most common mutation was missense (63/154, 40.9%), followed by large/complete deletions (50/154, 32.5%) and nonsense mutation (18/154, 11.7%). Frameshift, splice, and in-frame deletions/insertions occurred in 8.4% (13 of 154), 4.5% (7 of 154), and 1.3% (2 of 154) of patients, respectively (Table 1).

**Table 1:** Summary of the distribution of genetic mutations in the von Hippel–Lindau (*VHL*) gene between East Asian and Western patient cohorts

Type of *VHL* mutation	East Asian patients	Western patients [11] (%)
*Missense*	40.9% (63 of 154)	52.1
Exon 1	16.8% (26 of 154)	21.4
Exon 2	5.8% (9 of 154)	8.8
Exon 3	18.2% (28 of 154)	21.9
*Frameshift*	8.4% (13 of 154)	13.3
Exon 1	3.9% (6 of 154)	5.8
Exon 2	2.6% (4 of 154)	3.2
Exon 3	2.0% (3 of 154)	4.3
*Nonsense*	11.7% (18 of 154)	11.3
Exon 1	6.5% (10 of 154)	4.6
Exon 2	0% (0 of 154)	1.3
Exon 3	5.2% (8 of 154)	5.4
*Large/complete deletion*	32.5% (50 of 154)	10.8
Exon 1	5.2% (8 of 154)	1.1
Exon 2	3.2% (5 of 154)	0.4
Exon 3	5.8% (9 of 154)	0.3
Exons 1 and 2	0.6% (1 of 154)	0.3
Exons 1 and 3	0 (0 of 154)	0.2
Exons 2 and 3	1.3% (2 of 154)	0.4
Exons 1, 2 and 3	15.6% (24 of 154)	0.4
Exon not identified	0.6% (1 of 154)	6.8
Complete del	0% (0 of 154)	0.9
*Splice*	4.5% (7 of 154)	6.8
Exon 1	2.6% (4 of 154)	0.1
Exon 2	1.3% (2 of 154)	1.4
Exon 3	0.6% (1 of 154)	1.3
Location unknown	0%	4.0
*In-frame deletion/insertion*	1.3% (2 of 154)	5.6
Exon 1	1.3% (2 of 154)	5.1
Exon 2	0% (0 of 154)	0.2
Exon 3	0% (0 of 154)	0.3

In our study, missense mutations in the *VHL* gene were the most common; this was also the most common class of mutation reported by Nordstrom-O’Brien et al. [11]. Missense mutations were more common in the series reported by Nordstrom-O’Brien et al. [11], which comprised both East Asian and non-East Asian patients, than in our cohort (52.0% vs. 40.9%, *P* = 0.012). Frameshift mutations were more common in the series reported by Nordstrom-O’Brien et al. [11], as compared with our series of East Asian patients (13.0% vs. 8.4%, *P* < 0.001). In contrast, large/complete deletions were more common in our series (32.5% vs. 10.5%, *P* < 0.001).

We analyzed the distribution of exon deletions (Table 2) and compared this with the data collected by McNeill et al. [14] via the VHL Registry at Birmingham Women’s Hospital and the Department of Medical and Molecular Genetics, University of Birmingham, United Kingdom. No difference was found in the distribution of exon deletions. The most common mutation was a large deletion of exons 1, 2, and 3. Of those patients who had deletion of a single exon (22 of 50 similar mutations), exon 3 was the most commonly involved exon. No significant difference was observed in the distribution of exon deletions or in the risk of developing central nervous system hemangioblastoma, RCC, or RCH, irrespective of whether there was complete deletion of the *VHL* gene or incomplete deletion of the *VHL* gene (Table 3).

**Table 2:** Comparison of distribution of exon deletions in the *VHL* gene between East Asian and the cohort registered in Birmingham, United Kingdom

Deletion	East Asian cohort	McNeill et al. [14]	*P* value
Exon 1 AND (Exon 1 and FANCD2)	8 (16%)	8 (12.3%)	0.597
Exon 1 and 2	1 (2%)	4 (6.2%)	0.386
Exon 2	5 (10%)	12 (18.5%)	0.209
Exon 2 and 3	2 (4%)	1 (1.5%)	0.579
Exon 3	9 (18%)	15 (23.1%)	0.644
Exon 1 and 2 and 3 AND (Exon 1, 2, 3, and FANCD2)	24 (48%)	25 (38.4%)	0.349
Unknown	1 (2%)	0 (0%)	
Total	50 similar mutations	65 similar mutations	

All values are presented as the number of cases followed by the percentage in parentheses

The data of the cohort registered in Birmingham, United Kingdom was reported by McNeill et al. [14]

**Table 3:** Major complications of VHL syndrome and extent of deletion of the *VHL* gene

VHL syndrome	Complete deletion of the *VHL* gene (24 matches)	Incomplete deletion of the *VHL* gene (26 matches)
CNSH	23 (95.8%)	21 (80.8%)
RCH	4 (16.7%)	5 (19.2%)
RCC	16 (66.7%)	13 (50.0%)

*CNSH* central nervous system hemangioma, *RCH* retinal capillary hemangioblastoma, *RCC* renal cell carcinoma

**Fig. 1:**
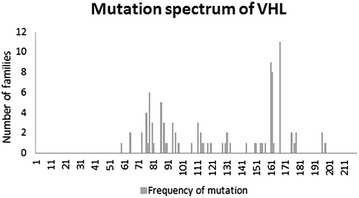
Frequency of point mutations in the von Hippel–Lindau (*VHL*) gene in an East Asian cohort

The most common single amino acid mutation was c.499 C>T (p.Arg167Trp), which was present in 4.5% (7 of 154) of patients (Fig. 1).

We found that 89.5% (17 of 19) of East Asian patients who developed PCC had an underlying missense mutation. The most frequently mutated residues in patients with PCC were Arg167 (36.8%, 7 of 19) and Arg161 (15.8%, 3 of 19),

### Phenotype

In our study of East Asian patients, we observed that the incidence of RCH was 27.9% (43 of 154). RCH was present in 38.1% (24 of 63) East Asian VHL kindreds with missense mutations, 2.5% (3 of 24) with complete exon deletions, and 19.2% (5 of 26) with exon deletions.

The overall frequency of RCC in this cohort was 57.8% (89 of 154). The frequency of RCC was 55.6% (10 of 18) in the families with nonsense mutations compared with 63.5% (40 of 63) in those with missense mutations (*P* = 0.541). A smaller proportion of families with frameshift mutations—46.2% (6 of 13 families)—developed RCC.

## Discussion

This study consolidated all published reports about VHL syndrome in East Asian patients for whom both genetic and clinical information were available, with which we could better understand the spectrum of VHL syndrome.

### PCCs associated with VHL syndrome

We found that 89.5% (17 of 19) of East Asian kindreds who developed PCC had an underlying missense mutation. This is consistent with the results in earlier studies that identified a missense mutation in the *VHL* gene in 83.5% to 96% of VHL patients who developed PCC [9, 11, 15]. The remaining 10.5% of patients in our study who developed PCC had a frameshift mutation. Again, these results are similar to those reported by Nordstrom-O’Brien et al. [11], who found that 7% of VHL type 2 families had frameshift mutations.

The most frequently mutated residues in this East Asian cohort were Arg167 (36.8%) and Arg161 (15.8%), which are both surface missense mutations. The Arg167 residue has been reported by Stebbins et al. [5] to have a structural role in stabilizing the H1 helix and the α-β domain interface. Forman et al. [16] used bioinformatics tools to determine that mutations at residues 161, 164, and 166 are at conserved sites with elongin C interactions and thus interfere with the elongin C interface. In our cohort, 3 of 4 matched patients who had the mutation Arg161Gln developed PCC at ages 10, 18, and 33 years. None of the 4 matched patients with a truncating mutation at Arg161 had PCC, which is consistent with our findings that most patients who develop PCC carry a missense mutation.

In our series, 12.3% of patients had type 2 VHL syndrome. In 1995, the Clinical Research Group for VHL in Japan reviewed data from Japan and reported that 9% of the evaluated patients had type 2 VHL syndrome [9]. A more recent nationwide epidemiologic survey of VHL syndrome in Japan reported a higher incidence of 15.1% for PCC [17]. Although improved screening may account for increased detection of PCC, another possibility for the increased incidence is age-dependent penetrance. The mean age at diagnosis of the first manifestation of VHL syndrome is between 24.7 and 26.3 years [4, 18], but almost complete penetrance is observed only by age 60 [1]. Thus, in a given population, some of the clinical manifestations that would develop over a lifetime may not have manifested yet.

### RCHs associated with VHL syndrome

The overall frequency of RCH in this East Asian cohort was 27.9% (43 of 154). In the literature, there is much variability in the reported frequency of RCH. Ong et al. [18] and Maher et al. [4] found the frequency of RCH to be 73% and 59%, respectively (Table 4). In a large cohort enrolled between 1988 and 2005 at the National Cancer Institute (National Institutes of Health, Bethesda, Maryland, USA), 890 patients with clinically definite VHL syndrome were screened, among them 335 (37.6%) were found to have ocular involvement with RCH [19].

**Table 4:** Comparison of frequency of various major complications of VHL syndrome across different cohorts

Clinical manifestation	Present study	Ong et al. [18]	Maher et al. [4]
CNSH	81.2% (125/154)	Cerebellar 57% Spinal 25%	Cerebellar 59% Spinal 13%
RCH	27.9% (43/154)	73%	59%
RCC	57.8% (89/154)	35%	28%
PCC	14.9% (23/154)	20%	7%

*CNSH* central nervous system hemangioblastoma, *RCH* retinal capillary hemangioblastoma, *RCC* renal cell carcinoma, *PCC* pheochromocytoma

We found that 38.1% (24 of 63) of East Asian VHL kindreds with missense mutations developed RCH, which is consistent with the findings of Mettu et al. [20], who found that 38.6% of 412 VHL patients with missense mutations had ocular VHL syndrome. In contrast, the prevalence of RCHs was lower in patients with exon deletions in the present study. RCHs were present in 12.5% (3 of 24) with complete exon deletions and in 19.2% (5 of 26) with exon deletions. This is similar to earlier findings that the prevalence of RCHs was lower in patients with complete deletions [21]. Interestingly, we found that RCHs were identified less frequently in East Asians; this may be because exon deletions are more common in East Asian patients with VHL syndrome. However, ascertainment bias in this series cannot be excluded since a comprehensive standard evaluation of affected individuals was not possible.

### RCCs associated with VHL syndrome

The overall frequency of RCC in this cohort was 57.8% (89 of 154), which was higher than that reported by Ong et al. [18] and Maher et al. [4] (35% and 28%, respectively). Previous studies suggested that truncating and frameshift mutations confer a higher risk of developing RCC compared with missense mutations (75% vs. 57%, *P* = 0.04) [22]. In this study, no significant difference was found: 55.6% (10 of 18 families) with nonsense mutations developed RCC compared with 63.5% (40 of 63 families) with missense mutations (*P* = 0.541). A smaller proportion of families with frameshift mutations—46.2% (6 of 13 families)—developed RCC. In this series, nearly two-thirds of patients with nonsense or missense mutations were subsequently diagnosed with RCC. For such patients, screening for RCC needs to be prioritized.

### Strengths and limitations

To minimize publication-related bias, articles in non-English language journals identified through the literature review were reviewed by native Chinese or Japanese speakers.

However, this study still had several limitations. An asymptomatic person can be identified as late as 67 years old, and only after careful screening [4]. As such, there may be under-estimation of the number of affected individuals, as some clinical manifestations may have been missed if they were not screened. Another limitation was that East Asian VHL patients who did not undergo genetic testing and lacked an identified genetic mutation were excluded from the analysis. Furthermore, duplicate data could not be completely excluded since patient identifiers were removed in reports, although we did our best to match identities across publications. The other constraint to accurately determining genotype-phenotype association was that the clinical workup of patients was not always disclosed; thus, when a person was not reported to have a particular manifestation, we could not determine whether this is because the investigations returned negative or the patient was asymptomatic and not investigated. We acknowledge that restricting our study selection to the PubMed database is a possible limitation; however, we do not expect bias in our conclusions, given the breadth of PubMed and that we included non-English language articles in our analysis.

## Conclusions

Evidence suggests that the genotypic and phenotypic characteristics of East Asian patients with VHL syndrome differ from other populations. This should be considered when making screening recommendations for VHL syndrome in Asia.

Patients with VHL syndrome are predisposed to a spectrum of benign and malignant tumors. As such, affected people should be thoroughly evaluated at regular intervals. However, since time and fiscal constraints remain very real considerations, physician’s familiarity with the genotypic and phenotypic profile of patients with VHL syndrome would enable judicious prioritization of screening.

## Additional file

10.1186/s40880-016-0141-z Genetic and clinical information about VHL disease in Asia.
