# Supplementary material for: Clinical and molecular characteristics of East Asian patients with von Hippel–Lindau syndrome
Source: Chin J Cancer. 2016 Aug 15;35:79. doi: 10.1186/s40880-016-0141-z (PMC4986176; doi:10.1186/s40880-016-0141-z)
Supplement: Supplementary file 1 — 10.1186/s40880-016-0141-z Genetic and clinical information about VHL disease in Asia. [file 40880_2016_141_MOESM1_ESM.docx]

| **Supporting table. Genetic and clinical information about VHL disease in Asia** | | | | | | | | | | |  |  |  |
| --- | --- | --- | --- | --- | --- | --- | --- | --- | --- | --- | --- | --- | --- |
| **S/N** | **cDNA** | **Predicted** | **Mutation type** | **CNSH** | **RCH** | **PCC** | **RCC** | **RC** | **PC** | **PT** | **ELST** | **Reference** | Ethnicity |
| 1 | c.173_174insC | p.Pro59fs | Frameshift | Y | N | N | N | N | N | N | N | [1] | Korean |
| 2 | c.194C>G | p.Ser65Trp | Missense | Y | N | N | Y | N | N | N | N | [2] | Chinese |
| 3 | c.194C>G | p.Ser65Trp | Missense | Y | Y | N | N | N | N | N | N | [3] | Chinese |
| 4 | c.208G>A | p.Glu70Lys | Missense | Y | N | N | N | N | N | N | N | [1] | Korean |
| 5 | c.217C>T | p.Gln73X | Nonsense | Y | N | N | Y | N | N | N | N | [1] | Korean |
| 6 | c.217C>T | p.Gln73X | Nonsense | N | N | N | Y | Y | Y | N | N | [4] | Chinese |
| 7 | c.223_224insT | p.Phe76fs | Frameshift | Y | Y | N | N | N | Y | Y | N | [5] | Korean |
| 8 | c.223_224insT | p.Phe76fs | Frameshift | Y | Y | N | Y | N | N | N | N | [1] | Korean |
| 9 | c.227_229del | p.Phe76del | Frameshift | Y | N | N | Y | N | N | N | N | [3] | Japanese |
| 10 | c.227_229delTCT | p.Phe76del | Inframe deletion | Y | N | N | Y | N | N | N | N | [2] | Chinese |
| 11 | c.230del | p.Cys77Serfs*82 | Frameshift | Y | N | N | Y | N | N | N | N | [3] | Japanese |
| 12 | c.232A>C | p.Asn78His | Missense | Y | Y | N | N | N | N | N | N | [3] | Japanese |
| 13 | c.233A>G | p.Asn78Ser | Missense | Y | N | N | Y | Y | Y | N | N | [4] | Chinese |
| 14 | c.233A>G | p.Asn78Ser | Missense | Y | N | N | Y | N | N | N | N | [6] | Chinese |
| 15 | c.233A>G | p.Asn78Ser | Missense | Y | N | N | N | N | N | N | N | [7] | Chinese |
| 16 | c.233A>G | p.Asn78Ser | Missense | Y | Y | N | N | N | N | N | N | [3] | Japanese |
| 17 | c.233A>T | p.Asn78Ile | Missense | Y | N | N | Y | N | Y | Y | N | [8] | Japanese |
| 18 | c.239del | p.Ser80Ilefs*79 | Frameshift | Y | Y | N | N | N | N | N | N | [3] | Chinese |
| 19 | c.239G>T | p.Ser80Ile | Missense | N | N | Y | Y | Y | Y | Y | N | [4] | Chinese |
| 20 | c.239G>T | p.Ser80Ile | Missense | Y | N | N | N | N | N | N | N | [6] | Chinese |
| 21 | c.241C>T | p.Pro81Ser | Missense | Y | N | N | N | N | N | N | N | [3] | Japanese |
| 22 | c.256C>T | p.Pro86Ser | Missense | N | Y | Y | Y | N | N | N | N | [2] | Chinese |
| 23 | c.256C>T | p.Pro86Ser | Missense | Y | Y | N | Y | N | N | N | N | [3] | Japanese |
| 24 | c.257C>T | p.Pro86Leu | Missense | N | N | N | Y | N | N | N | N | [4] | Chinese |
| 25 | c.257C>T | p.Pro86Leu | Missense | Y | Y | N | N | N | Y | N | N | [9] | Japanese |
| 26 | c.257C>T | p.Pro86Leu | Missense | N | Y | N | N | N | N | N | N | [3] | Japanese |
| 27 | c.263G>A | p.Trp88X | Nonsense | Y | N | N | Y | N | N | N | N | [2] | Chinese |
| 28 | c.263G>A | p.Trp88X | Nonsense | N | N | N | Y | N | N | N | N | [2] | Chinese |
| 29 | c.263G>C | p.Trp88Ser | Missense | N | N | N | Y | N | N | N | N | [3] | Japanese |
| 30 | c.265C>T | p.Leu89Ser | Missense | N | Y | N | Y | Y | N | N | N | [4] | Chinese |
| 31 | c.269A>T | p.Asn90Ile | Missense | Y | N | N | Y | N | N | N | N | [2] | Chinese |
| 32 | c.280G>T | p.Glu94X | Nonsense | Y | N | N | Y | N | N | N | N | [2] | Chinese |
| 33 | c.280G>T | p.Glu94X | Nonsense | Y | N | N | N | N | N | N | N | [2] | Chinese |
| 34 | c.280G>T | p.Glu94X | Nonsense | Y | N | N | N | N | Y | N | N | [10] | Chinese |
| 35 | c.286C>T | p.Gln96X | Nonsense | Y | N | N | Y | N | N | N | N | [4] | Chinese |
| 36 | c.288_290del | p.Gln96_Pro97delinsHis | Inframe deletion | N | Y | N | Y | N | N | N | N | [3] | Japanese |
| 37 | c.293A>G | p.Tyr98Cys | Missense | Y | N | Y | Y | N | N | N | N | [11]. | Japanese |
| 38 | c.319C>G | p.Arg107Gly | Missense | Y | N | N | Y | N | N | N | N | [6] | Chinese |
| 39 | c.332G>A | p.Ser111Asn | Missense | Y | Y | N | Y | Y | Y | N | N | [4] | Chinese |
| 40 | c.333C>A | p.Ser111Arg | Missense | Y | N | N | Y | N | Y | Y | N | [12] | Japanese |
| 41 | c.333C>A | p.Ser111Arg | Missense | Y | N | N | N | N | N | N | N | [13] | Japanese |
| 42 | c.337C>T | p. Arg113X | Nonsense | Y | N | N | Y | Y | Y | Y | N | [14] |  |
| 43 | c.337C>T | p.Arg113X | Nonsense | Y | N | N | N | N | N | N | N | [15] | Taiwan people |
| 44 | c.340+1G>A | - | to A on a splicing site at position 553 + 1 | N | N | N | N | N | N | N | Y | [16] |  |
| 45 | c.340+1G>A | - | Splice site mutation | N | N | N | N | N | N | N | Y | [17] | Chinese |
| 46 | c.340+1G>A | - | Splice site mutation | Y | N | N | N | N | N | N | N | [3] | Chinese |
| 47 | c.340+1G>C | - | Splice site mutation | Y | N | N | N | N | N | Y | N | [3] | Chinese |
| 48 | c.340+5G>C | - | Splice site mutation | Y | N | N | N | N | N | N | N | [18] | Japanese |
| 49 | c.340G>C | p.Gly114Arg | Missense | Y | N | Y | Y | Y | Y | Y | N | Local data | Chinese |
| 50 | c.344A>G | p.His115Arg | Missense | Y | N | N | Y | N | N | N | N | [2] | Chinese |
| 51 | c.353T>C | p.Leu118Pro | Missense | N | N | N | Y | N | N | N | N | [3] | Japanese |
| 52 | c.358_359insAC | p.Arg120Asnfs*40 | Frameshift | N | N | N | Y | N | N | N | N | [3] | Japanese |
| 53 | c.383T>C | p.Leu128Pro | Missense | Y | N | N | Y | N | N | N | N | [1] | Korean |
| 54 | c.388G>T | p.Val130Phe | Missense | Y | N | N | N | N | N | Y | N | [3] | Chinese |
| 55 | c.392A>G | p.Asn131Ser | Missense | Y | Y | Y | N | N | N | N | N | [19] | Japanese |
| 56 | c.393C>G | p.Asn131Lys | Missense | Y | Y | N | Y | Y | Y | N | N | Local data | Chinese |
| 57 | c.397_398insA | p.Thr133Asnfs | Frameshift | Y | N | N | N | N | N | N | N | [15] | Taiwan people |
| 58 | c.429_439del | p.Gly144Phefs*26 | Frameshift | Y | Y | N | Y | N | N | N | N | [3] | Japanese |
| 59 | c.449del | p.Asn150Ilefs*9 | Frameshift | N | N | N | Y | Y | Y | N | N | [4] | Chinese |
| 60 | c.451A>T | p.Ile151Phe | Missense | N | N | N | Y | N | N | N | Y | [2] | Chinese |
| 61 | c.460C>T | p.Pro154Ser | Missense | N | N | Y | Y | N | N | N | N | [20] | Japanese |
| 62 | c.463+1G>T | - | Splice site mutation | Y | N | N | Y | N | N | N | N | [6] | Chinese |
| 63 | c.463G>A | p.Val155Met | Missense | Y | N | N | Y | N | N | N | N | [3] | Japanese |
| 64 | c.464-1G>T | - | Splice site mutation | Y | Y | N | Y | N | N | N | N | [3] | Japanese |
| 65 | c.470C>T | p.Thr157Ile | Missense | N | N | Y | Y | N | N | N | N | [3] | Japanese |
| 66 | c.481 C>T | p.Arg161X | Nonsense | Y | N | N | N | N | N | N | N | [21] | Japanese |
| 67 | c.481C>A | p.Arg161Arg | Missense | Y | N | N | Y | N | N | N | N | [2] | Chinese |
| 68 | c.481C>T | p.Arg161X | Nonsense | Y | Y | N | Y | Y | Y | N | N | Local data | Malay |
| 69 | c.481C>T | p.Arg161X | Nonsense | Y | N | N | Y | N | N | N | N | [6] | Chinese |
| 70 | c.481C>T | p.Arg161X | Nonsense | N | Y | N | N | N | N | N | N | [1] | Korean |
| 71 | c.482G>A | p.Arg161Gln | Missense | Y | Y | Y | N | N | N | N | N | [22] | Japanese |
| 72 | c.482G>A | p.Arg161Gln | Missense | Y | Y | N | N | N | N | Y | N | [23] | Chinese |
| 73 | c.482G>A | p.Arg161Gln | Missense | N | N | Y | N | N | N | N | N | [6] | Chinese |
| 74 | c.482G>A | p.Arg161Gln | Missense | N | N | Y | N | N | N | N | N | [6] | Chinese |
| 75 | c.484T>C | p.Cys162Arg | Missense | Y | Y | N | Y | N | N | N | N | [1] | Korean |
| 76 | c.484T>C | p.Cys162Arg | Missense | Y | Y | N | Y | Y | Y | Y | N | [5] | Korean |
| 77 | c.484T>C | p.Cys162Arg | Missense | Y | Y | N | Y | N | N | N | N | [3] | Japanese |
| 78 | c.485G>A | p.Cys162Tyr | Missense | Y | N | N | N | N | N | N | N | [3] | Japanese |
| 79 | c.486C>G | p.Cys162Trp | Missense | Y | N | N | Y | N | Y | N | N | [24] | Japanese |
| 80 | c.486C>G | p.Cys162Trp | Missense | Y | N | N | Y | Y | N | N | N | [4] | Chinese |
| 81 | c.486C>G | p.Cys162Trp | Missense | Y | N | N | N | N | N | N | N | [1] | Korean |
| 82 | c.486C>G | p.Cys162Trp | Missense | Y | N | N | N | Y | Y | N | N | [5] | Korean |
| 83 | c.488T>C | p.Leu163Pro | Missense | Y | N | N | Y | N | N | N | N | [1] | Korean |
| 84 | c.499C>T | p.Arg167Trp | Missense | Y | N | N | Y | N | N | N | Y | [2] | Chinese |
| 85 | c.499C>T | p.Arg167Trp | Missense | Y | Y | Y | Y | N | N | N | N | [3] | Japanese |
| 86 | c.499C>T | p.Arg167Trp | Missense | Y | Y | Y | Y | N | N | N | N | [3] | Japanese |
| 87 | c.499C>T | p.Arg167Trp | Missense | N | N | N | Y | N | N | Y | N | [25] | Japanese |
| 88 | c.499C>T | p.Arg167Trp | Missense | Y | N | Y | Y | N | N | N | N | [6] | Chinese |
| 89 | c.499C>T | p.Arg167Trp | Missense | N | Y | Y | N | N | N | Y | N | Local data | Chinese |
| 90 | c.499C>T | p.Arg167Trp | Missense | Y | Y | Y | N | N | N | Y | N | [26] | Japanese |
| 91 | c.500G>A | p.Arg167Gln | Missense | Y | Y | Y | Y | N | N | N | N | [27] | Japanese |
| 92 | c.500G>A | p.Arg167Gln | Missense | Y | Y | N | Y | N | N | N | N | [3] | Japanese |
| 93 | c.500G>A | p.Arg167Gln | Missense | Y | N | N | N | N | N | N | N | [3] | Chinese |
| 94 | c.500G>A | p.Arg167Gln | Missense | Y | N | Y | N | N | N | Y | N | [28] | Japanese |
| 95 | c.522delT | p.Tyr175Thrfs | Frameshift | Y | N | N | N | N | N | N | N | [15] | Taiwan people |
| 96 | c.525C>G | p.Tyr175X | Nonsense | Y | N | N | N | Y | Y | N | N | Local data | Chinese |
| 97 | c.529A>T | p.Arg177X | Nonsense | Y | Y | N | N | N | N | N | N | [3] | Chinese |
| 98 | c.533T>C | p.Leu178Pro | Missense | Y | Y | N | Y | N | N | N | N | [3] | Japanese |
| 99 | c.533T>G | p.Leu178Arg | Missense | Y | Y | N | Y | N | N | N | N | [2] | Chinese |
| 100 | c.586A>T | p.Lys196X | Nonsense | Y | N | N | Y | Y | Y | Y | N | [5] | Korean |
| 101 | c.586A>T | p.Lys196X | Nonsense | Y | N | N | N | N | N | N | N | [1] | Korean |
| 102 | c.593_613dup | p.Leu198_Glu204dup | Frameshift | Y | Y | Y | N | N | N | N | N | [29] | Japanese |
| 103 | c.776_777ins20 | - | Frameshift | Y | Y | Y | N | N | N | N | N | [3] | Japanese |
| 104 | c.*98G>T | - | Mutation in 5' UTR location | N | N | N | N | Y | N | N | N | [30] | Chinese |
| 105 | Exon 1 and 2 del | Exon 1 and 2 del | Exon del | Y | N | N | Y | N | N | N | N | [31] | Japanese |
| 106 | Exon 1 del | Exon 1 del | Exon del | Y | N | N | N | N | N | N | N | [32] | Chinese |
| 107 | Exon 1 del | Exon 1 del | Exon del | Y | N | N | N | N | N | N | N | [32] | Chinese |
| 108 | Exon 1 del | Exon 1 del | Exon del | Y | Y | N | N | N | N | N | N | [32] | Chinese |
| 109 | Exon 1 del | Exon 1 del | Exon del | N | Y | N | N | N | N | N | N | [32] | Chinese |
| 110 | Exon 1 del | Exon 1 del | Exon del | Y | Y | N | N | N | N | N | N | [32] | Chinese |
| 111 | Exon 1 del | Exon 1 del | Exon del | Y | N | N | Y | N | N | N | N | [31] | Japanese |
| 112 | Exon 1 del | Exon 1 del | Exon del | N | N | Y | N | N | N | N | N | [31] | Japanese |
| 113 | Exon 1 del | Exon 1 del | Exon del | N | N | Y | N | N | N | N | N | [31] | Japanese |
| 114 | Exon 1, 2 and 3 del | Exon 1, 2 and 3 del | Exon 1, 2 and 3 del | Y | N | N | Y | N | N | N | N | [1] | Korean |
| 115 | Exon 1, 2 and 3 del | Exon 1, 2 and 3 del | Exon 1, 2 and 3 del | Y | N | N | Y | N | N | N | N | [1] | Korean |
| 116 | Exon 1, 2 and 3 del | Exon 1, 2 and 3 del | Exon 1, 2 and 3 del | Y | N | N | Y | N | N | N | N | [1] | Korean |
| 117 | Exon 1, 2 and 3 del | Exon 1, 2 and 3 del | Exon 1, 2 and 3 del | Y | N | N | Y | N | N | N | N | [3] | Japanese |
| 118 | Exon 1, 2 and 3 del | Exon 1, 2 and 3 del | Exon 1, 2 and 3 del | Y | N | N | Y | N | N | N | N | [3] | Japanese |
| 119 | Exon 1, 2 and 3 del | Exon 1, 2 and 3 del | Exon 1, 2 and 3 del | Y | N | N | N | N | N | N | N | [32] | Chinese |
| 120 | Exon 1, 2 and 3 del | Exon 1, 2 and 3 del | Exon 1, 2 and 3 del | Y | Y | N | N | N | Y | N | N | [32] | Chinese |
| 121 | Exon 1, 2 and 3 del | Exon 1, 2 and 3 del | Exon 1, 2 and 3 del | Y | N | N | N | N | N | N | N | [1] | Korean |
| 122 | Exon 1, 2 and 3 del | Exon 1, 2 and 3 del | Exon 1, 2 and 3 del | Y | N | N | N | N | N | N | N | [1] | Korean |
| 123 | Exon 1, 2 and 3 del | Exon 1, 2 and 3 del | Exon 1, 2 and 3 del | Y | Y | N | N | N | N | N | N | [3] | Japanese |
| 124 | Exon 1, 2 and 3 del | Exon 1, 2 and 3 del | Exon 1, 2 and 3 del | Y | N | N | N | Y | N | N | N | [31] | Chinese |
| 125 | Exon 1, 2 and 3 del | Exon 1, 2 and 3 del | Exon 1, 2 and 3 del | Y | N | N | N | Y | N | N | N | [31] | Japanese |
| 126 | Exon 1, 2 and 3 del | Exon 1, 2 and 3 del | Exon 1, 2 and 3 del | Y | N | N | Y | N | N | N | N | [31] | Japanese |
| 127 | Exon 1, 2 and 3 del | Exon 1, 2 and 3 del | Exon 1, 2 and 3 del | N | Y | N | N | Y | N | N | N | [31] | Japanese |
| 128 | Exon 1, 2 and 3 del | Exon 1, 2 and 3 del | Exon 1, 2 and 3 del | Y | N | N | Y | N | N | N | N | [31] | Japanese |
| 129 | Exon 1, 2 and 3 del | Exon 1, 2 and 3 del | Exon 1, 2 and 3 del | Y | N | N | Y | N | N | N | N | [31] | Japanese |
| 130 | Exon 1, 2 and 3 del | Exon 1, 2 and 3 del | Exon 1, 2 and 3 del | Y | N | N | Y | N | N | N | N | [31] | Japanese |
| 131 | Exon 1, 2 and 3 del | Exon 1, 2 and 3 del | Exon 1, 2 and 3 del | Y | N | N | Y | N | N | N | N | [31] | Japanese |
| 132 | Exon 1, 2 and 3 del | Exon 1, 2 and 3 del | Exon 1, 2 and 3 del | Y | N | N | Y | N | N | N | N | [31] | Japanese |
| 133 | Exon 1, 2 and 3 del | Exon 1, 2 and 3 del | Exon 1, 2 and 3 del | Y | N | Y | Y | N | N | N | N | [31] | Japanese |
| 134 | Exon 1, 2 and 3 del | Exon 1, 2 and 3 del | Exon 1, 2 and 3 del | Y | N | N | Y | N | N | N | N | [31] | Japanese |
| 135 | Exon 1, 2 and 3 del | Exon 1, 2 and 3 del | Exon 1, 2 and 3 del | Y | N | N | Y | N | N | N | N | [31] | Japanese |
| 136 | Exon 1, 2 and 3 del | Exon 1, 2 and 3 del | Exon 1, 2 and 3 del | Y | N | N | Y | Y | N | N | N | [31] | Japanese |
| 137 | Exon 1, 2 and 3 del | Exon 1, 2 and 3 del | Exon 1, 2 and 3 del | Y | N | N | Y | Y | N | N | N | [31] | Japanese |
| 138 | Exon 2 and 3 del | Exon 2 and 3 del | Exon del | Y | N | N | N | Y | Y | N | N | [5] | Korean |
| 139 | Exon 2 and 3 del | Exon 2 and 3 del | Exon del | Y | N | N | Y | N | N | N | N | [31] | Japanese |
| 140 | Exon 2 del | Exon 2 del | Exon del | Y | N | N | Y | Y | Y | N | N | Local data | Chinese |
| 141 | Exon 2 del | Exon 2 del | Exon del | Y | N | N | Y | N | N | N | N | [6] | Chinese |
| 142 | Exon 2 del | Exon 2 del | Exon del | Y | N | N | Y | N | N | N | N | [31] | Japanese |
| 143 | Exon 2 del | Exon 2 del | Exon del | Y | N | N | Y | N | N | N | N | [31] | Japanese |
| 144 | Exon 2 del | Exon 2 del | Exon del | Y | N | N | Y | N | N | N | N | [31] | Japanese |
| 145 | Exon 3 del | Exon 3 del | Exon del | N | N | N | Y | Y | Y | Y | N | Local data | Chinese |
| 146 | Exon 3 del | Exon 3 del | Exon del | Y | Y | N | Y | N | N | N | N | [2] | Chinese |
| 147 | Exon 3 del | Exon 3 del | Exon del | Y | Y | N | Y | N | N | N | N | [2] | Chinese |
| 148 | Exon 3 del | Exon 3 del | Exon del | Y | N | N | N | N | N | N | N | [32] | Chinese |
| 149 | Exon 3 del | Exon 3 del | Exon del | Y | N | N | N | N | N | N | N | [32] | Chinese |
| 150 | Exon 3 del | Exon 3 del | Exon del | Y | N | N | N | N | N | N | N | [32] | Chinese |
| 151 | Exon 3 del | Exon 3 del | Exon del | Y | N | N | N | N | N | N | N | [32] | Chinese |
| 152 | Exon 3 del | Exon 3 del | Exon del | N | N | Y | Y | N | N | N | N | [31] | Japanese |
| 153 | Exon 3 del | Exon 3 del | Exon del | Y | N | N | Y | N | N | N | N | [31] | Japanese |
| 154 | Exon del (unknown) | Exon del (unknown) | Exon del | Y | N | N | N | N | N | N | N | [15] | Taiwan people |
|  |  |  |  |  |  |  |  |  |  |  |  |  |  |
|  | *Legend:* | |  |  |  |  |  |  |  |  |  |  |  |
|  | *CNSH :* | *Central nervous system haemangioblastoma* | | |  |  |  |  |  |  |  |  |  |
|  | *RA:* | *Retinal capillary haemangioblastoma* | | |  |  |  |  |  |  |  |  |  |
|  | *PCC:* | *Phaechromocytoma* | | |  |  |  |  |  |  |  |  |  |
|  | *RCC:* | *Renal cell carcinoma* | | |  |  |  |  |  |  |  |  |  |
|  | *RC:* | *Renal cyst* | | |  |  |  |  |  |  |  |  |  |
|  | *PC:* | *Pancreatic cyst* | | |  |  |  |  |  |  |  |  |  |
|  | *PT:* | *Pancreat tumour* | | |  |  |  |  |  |  |  |  |  |
|  | *ELST:* | *Endolymphatic sac tumour* | | |  |  |  |  |  |  |  |  |  |

Footnote: Families 56, 89, 96, and 140 are patients who have been diagnosed in Singapore, and have not been published hitherto thus there is no available reference.

References

| 1 | Cho HJ, Ki CS, Kim JW. Improved detection of germline mutations in Korean VHL patients by multiple ligation-dependent probe amplification analysis. Journal of Korean medical science. 2009 Feb;24(1):77-83. PubMed PMID: 19270817. Pubmed Central PMCID: PMC2650969. Epub 2009/03/10. eng. |
| --- | --- |
| 2 | Wu P, Zhang N, Wang X, Ning X, Li T, Bu D, et al. Family history of von Hippel-Lindau disease was uncommon in Chinese patients: suggesting the higher frequency of de novo mutations in VHL gene in these patients. Journal of human genetics. 2012 Apr;57(4):238-43. PubMed PMID: 22357542. Epub 2012/02/24. eng. |
| 3 | Zhou J, Wang J, Li N, Zhang X, Zhou H, Zhang R, et al. Molecularly genetic analysis of von Hippel-Lindau associated central nervous system hemangioblastoma. Pathology international. 2010 Jun;60(6):452-8. PubMed PMID: 20518900. Epub 2010/06/04. eng. |
| 4 | Zhang J, Huang YR, Liu DM, Zhou LX, Xue W, Chen Q, et al. Management of solid renal tumour associated with von Hippel-Lindau disease. Chinese medical journal. 2007 Nov 20;120(22):2049-52. PubMed PMID: 18067796. Epub 2007/12/11. eng. |
| 5 | Lee KH, Lee JS, Kim BJ, Lee JK, Kim SH, Kim SH, et al. Pancreatic involvement in Korean patients with von Hippel-Lindau disease. Journal of gastroenterology. 2009;44(5):447-52. PubMed PMID: 19333546. Epub 2009/04/01. eng. |
| 6 | Siu WK, Ma RC, Lam CW, Mak CM, Yuen YP, Lo FM, et al. Molecular basis of von Hippel-Lindau syndrome in Chinese patients. Chinese medical journal. 2011 Jan;124(2):237-41. PubMed PMID: 21362373. Epub 2011/03/03. eng. |
| 7 | Zhang J, Huang YR, Wang JD, Fan XD. [Familial and genetic study in a large Chinese kindred with von Hippel-Lindau disease and gene mutation analysis]. Zhonghua yi xue yi chuan xue za zhi = Zhonghua yixue yichuanxue zazhi = Chinese journal of medical genetics. 2004 Feb;21(1):5-9. PubMed PMID: 14767899. Epub 2004/02/10. chi. |
| 8 | Kanno A, Satoh K, Hamada S, Hirota M, Masamune A, Motoi F, et al. Serous cystic neoplasms of the whole pancreas in a patient with von Hippel-Lindau disease. Internal medicine (Tokyo, Japan). 2011;50(12):1293-8. PubMed PMID: 21673464. Epub 2011/06/16. eng. |
| 9 | Fukino K, Teramoto A, Adachi K, Takahashi H, Emi M. A family with hydrocephalus as a complication of cerebellar hemangioblastoma: identification of Pro157Leu mutation in the VHL gene. Journal of human genetics. 2000;45(1):47-51. PubMed PMID: 10697963. Epub 2000/03/04. eng. |
| 10 | Sun Y, Sun Q, Shen J, Wu H, Guan Y, Gong K, et al. Cauda equina hemangioblastoma at L5 vertebral level related to von Hippel-Lindau disease. British journal of neurosurgery. 2012 Aug;26(4):576-7. PubMed PMID: 22133049. Epub 2011/12/03. eng. |
| 11 | Arao T, Okada Y, Tanikawa T, Inatomi H, Shuin T, Fujihira T, et al. A case of von Hippel-Lindau disease with bilateral pheochromocytoma, renal cell carcinoma, pelvic tumor, spinal hemangioblastoma and primary hyperparathyroidism. Endocrine journal. 2002 Apr;49(2):181-8. PubMed PMID: 12081237. Epub 2002/06/26. eng. |
| 12 | Maeda H, Okabayashi T, Kobayashi M, Araki K, Kohsaki T, Nishimori I, et al. Emergency pancreatoduodenectomy for pancreatic metastasis from renal cell carcinoma in a patient with von Hippel-Lindau disease: a case report. Digestive diseases and sciences. 2006 Aug;51(8):1383-7. PubMed PMID: 16868829. Epub 2006/07/27. eng. |
| 13 | Nakashima H, Tokunaga K, Tamiya T, Matsumoto K, Ohmoto T, Furuta T. [Analysis of spinal cord hemangioblastoma in von Hippel-Lindau disease]. No shinkei geka Neurological surgery. 1999 Jun;27(6):533-40. PubMed PMID: 10396736. Epub 1999/07/09. jpn. |
| 14 | Osawa A, Sumiyama Y, Watanabe M, Tanaka H, Asai K, Enomoto T, et al. Single case of renal cell carcinoma and endocrine pancreatic head cancer occurring with von Hippel-Lindau disease. Journal of hepato-biliary-pancreatic surgery. 2006;13(2):174-80. PubMed PMID: 16547682. Epub 2006/03/21. eng. |
| 15 | Huang JS, Lin CM, Cheng YC, Hung KL, Chien CC, Chen SK, et al. A vitronectin M381T polymorphism increases risk of hemangioblastoma in patients with VHL gene defect. Journal of molecular medicine (Berlin, Germany). 2009 Jun;87(6):613-22. PubMed PMID: 19288063. Epub 2009/03/17. eng. |
| 16 | Rao, Q., Zhou, J., Wang, J. D., Jin, X. Z., Ma, H. H., Lu, Z. F., & Zhou, X. J. (2010). Endolymphatic sac tumor with von Hippel-Lindau disease: report of a case with analysis of von Hippel-Lindau gene and review. Ann Diagn Pathol, 14(5), 361-364. doi: 10.1016/j.anndiagpath.2009.10.001 |
| 17 | Rao, Q., Zhou, X. J., Jin, X. Z., Ma, H. H., Zhou, H. B., & Lu, Z. F. (2010). [Clinicopathological features and molecular genetic analysis of endolymphatic sac tumor: report of 2 cases]. Zhonghua Bing Li Xue Za Zhi, 39(6), 412-413. |
| 18 | Karasaki H, Ishizaki A, Yanagawa N, Nakano Y, Sasajima J, Mizukami Y, et al. [Two cases of pancreatic tumor with von Hippel-Lindau disease]. Nihon Shokakibyo Gakkai zasshi = The Japanese journal of gastro-enterology. 2008 May;105(5):725-31. PubMed PMID: 18460863. Epub 2008/05/08. jpn. |
| 19 | Imanaka M, Iida K, Takahashi K, Tsuji K, Nishizawa H, Fukuoka H, et al. The N131S mutation in the von Hippel-Lindau gene in a Japanese family with pheochromocytoma and hemangioblastomas. Endocrine journal. 2006 Dec;53(6):819-27. PubMed PMID: 17001110. Epub 2006/09/27. eng. |
| 20 | Takahashi K, Iida K, Okimura Y, Takahashi Y, Naito J, Nishikawa S, et al. A novel mutation in the von Hippel-Lindau tumor suppressor gene identified in a Japanese family with pheochromocytoma and hepatic hemangioma. Internal medicine (Tokyo, Japan). 2006;45(5):265-9. PubMed PMID: 16595991. Epub 2006/04/06. eng. |
| 21 | Sora S, Ueki K, Saito N, Kawahara N, Shitara N, Kirino T. Incidence of von Hippel-Lindau disease in hemangioblastoma patients: the University of Tokyo Hospital experience from 1954-1998. Acta neurochirurgica. 2001 Sep;143(9):893-6. PubMed PMID: 11685621. Epub 2001/10/31. eng. |
| 22 | Goto T, Nishi T, Kunitoku N, Yamamoto K, Kitamura I, Takeshima H, et al. Suprasellar hemangioblastoma in a patient with von Hippel-Lindau disease confirmed by germline mutation study: case report and review of the literature. Surgical neurology. 2001 Jul;56(1):22-6. PubMed PMID: 11546565. Epub 2001/09/08. eng. |
| 23 | Tong AL, Zeng ZP, Zhou YR, Yuan T, Cao CX, Zhang J, et al. Bilateral pheochromocytoma as first presentation of von Hippel-Lindau disease in a Chinese family. Chinese medical sciences journal = Chung-kuo i hsueh k'o hsueh tsa chih / Chinese Academy of Medical Sciences. 2009 Dec;24(4):197-201. PubMed PMID: 20120764. Epub 2010/02/04. eng. |
| 24 | Kume H, Kameyama S, Tanaka Y, Kitamura T. Cerebellar hemangioblastoma as a late manifestation of sporadic von Hippel-Lindau disease. The Journal of urology. 1999 Mar;161(3):911-2. PubMed PMID: 10022713. Epub 1999/02/18. eng. |
| 25 | Akatsu T, Aiura K, Ito Y, Ueda M, Kameyama K, Kitajima M. A novel Von Hippel-Lindau case with germline mutation at codon 167 (CGG to TGG) having endocrine microadenomatosis of the pancreas. Digestive diseases and sciences. 2007 Nov;52(11):3145-8. PubMed PMID: 17406817. Epub 2007/04/05. eng. |
| 26 | Matsuo T, Himei K, Ichimura K, Yanai H, Nose S, Mimura T, et al. Long-term effect of external beam radiotherapy of optic disc hemangioma in a patient with von Hippel-Lindau disease. Acta medica Okayama. 2011 Apr;65(2):135-41. PubMed PMID: 21519372. Epub 2011/04/27. eng. |
| 27 | Wu Y, Nishio H, Lee MJ, Ayaki H, Hayashi A, Ooba T, et al. Molecular genetic analysis and mutation screening of the VHL gene in a Japanese family with von Hippel-Lindau disease. The Kobe journal of medical sciences. 2000 Aug;46(4):147-53. PubMed PMID: 11354926. Epub 2001/05/17. eng. |
| 28 | Tomita N, Moriguchi A, Yamasaki K, Taniyama Y, Kotani N, Hashiya N, et al. A family with von Hippel-Lindau disease revealed by pheochromocytoma. Hypertension research : official journal of the Japanese Society of Hypertension. 2001 Jul;24(4):445-50. PubMed PMID: 11510758. Epub 2001/08/21. eng. |
| 29 | Miyagawa Y, Nakazawa M, Noda Y, Ito S, Ohguro H. von Hippel-Lindau disease type 2A in a family with a duplicated 21-base-pair in-frame insertion mutation in the VHL gene. Graefe's archive for clinical and experimental ophthalmology = Albrecht von Graefes Archiv fur klinische und experimentelle Ophthalmologie. 2003 Mar;241(3):241-4. PubMed PMID: 12644949. Epub 2003/03/20. eng. |
| 30 | Jin W, Wang LM, Zheng JF, Li Z, Huang YR. [The detection of mutations in VHL gene from a single cell in a patient with von Hippel-Lindau disease]. Zhonghua yi xue yi chuan xue za zhi = Zhonghua yixue yichuanxue zazhi = Chinese journal of medical genetics. 2007 Feb;24(1):67-71. PubMed PMID: 17285548. Epub 2007/02/08. chi. |
| 31 | Hattori K, Teranishi J, Stolle C, Yoshida M, Kondo K, Kishida T, et al. Detection of germline deletions using real-time quantitative polymerase chain reaction in Japanese patients with von Hippel-Lindau disease. Cancer science. 2006 May;97(5):400-5. PubMed PMID: 16630138. Epub 2006/04/25. eng. |
| 32 | Zhang J, Chen HG, Xue W, Zhou LX, Huang YR. [Large germline deletion of the VHL gene in Chinese families with von Hippel-Lindau syndrome]. Zhonghua yi xue yi chuan xue za zhi = Zhonghua yixue yichuanxue zazhi = Chinese journal of medical genetics. 2009 Oct;26(5):539-41. PubMed PMID: 19806577. Epub 2009/10/07. chi. |
